# Evidence of WNV infection in migratory birds passing through Xinjiang, China, using viral genome amplicon approach

**DOI:** 10.3389/fmicb.2025.1468530

**Published:** 2025-03-26

**Authors:** Kunsheng Tao, Chan He, Tong Zhang, Changguang Xiao, Lifei Du, Zongjie Li, Donghua Shao, Jianchao Wei, Beibei Li, Yafeng Qiu, Zhiyong Ma, Ke Liu

**Affiliations:** ^1^Shanghai Veterinary Research Institute, Chinese Academy of Agricultural Science, Shanghai, China; ^2^Hunan Institute of Animal and Veterinary Science, Changsha, China

**Keywords:** West Nile virus, migratory bird, amplicon, fecal samples, epidemiology

## Abstract

The West Nile virus (WNV) is a mosquito-borne virus of the Flaviviridae family that is transmitted through the mosquito-migratory bird-mosquito cycle. Currently, WNV infection is widespread in the Americas, Europe, and Africa, and is one of the most important global epidemic infectious diseases. Although migratory birds play an important role in the spread of WNV, monitoring of migratory birds carrying the WNV remains limited. Here, we developed a new nucleic acid test for detecting migratory birds carrying WNV, which uses amplicons of WNV to test fecal samples from migratory birds. This new method was validated by using full-length WNV genomic plasmid. With this amplicon method, we tested the migratory bird droppings collected in different locations. The results indicated that the positive rate of WNV nucleic acid in migratory bird droppings was over 39%, which provides clues to the fact that migratory birds may carry the WNV in Xinjiang, China.

## 1 Introduction

WNV is primarily transmitted through the bite of infected mosquitoes (Olufemi et al., 2021; Ronca et al., 2021). Mosquito become infected by feeding on birds that carry the virus. In nature, WNV is maintained in transmission cycle between birds and ornithophilic mosquitoes (Ferraguti et al., 2024). Mosquito species such as members of the *Culex pipiens* complex and *Aedes* spp. can act as bridge vectors and transmit WNV to vertebrate host species, including humans and horses. Virus-carrying birds migrate to various areas seasonally, and mosquitoes in the local area may be infected with the WNV by biting and sucking blood of the birds. Subsequently, infected mosquitoes may transmit the virus to other hosts by biting humans or other animals. Thus, the migratory bird-mosquito cycle is the limiting factor in the occurrence of regional epidemics of West Nile fever virus, which may result in regional epidemics when virus-carrying mosquitoes bite people or animals (Seidowski et al., 2010; Li et al., 2013).

Human infection with WNV is usually asymptomatic (>80%), and of those infected, ~20% will develop to West Nile fever, which manifests as fever, diarrhea, and respiratory symptoms, easily confused with a cold, and lasts for 3–6 days. Less than 1% of these infected individuals develop severe neuroinvasive disease, which is characterized by three clinical signs: West Nile meningitis, West Nile encephalitis, and acute flaccid paralysis (Klingelhöfer et al., 2023). The number of neuroinvasive cases and deaths increases with age, especially in people aged 65–89 years. The risk of neuroinvasive disease is nearly 1/50 in the elderly population aged 65 years or older, and is 16 times higher than that of people aged 16–24 years, with a median age of 64 years for neuroinvasive cases and 49 years for West Nile fever cases. Like humans, mammals such as horses and cattle are terminal hosts for WNV, and infection results in neuroinvasive diseases including encephalitis, fatal encephalomyelitis, and abortion in pregnant horses. In horses, for example, ~8% of horses infected with WNV develop severe neurological signs, with ataxia as the primary clinical manifestation and progression to encephalomyelitis. Other common signs include weakness of the limbs, lateral recumbency, and muscle tremors. Susceptible birds are storage and amplification hosts for WNV. Migratory birds infected with WNV mainly show neurological and respiratory symptoms including weakness, paralysis, visual disturbances, respiratory distress and runny nose, etc. Infected birds develop viraemia in their bodies and lead to death, with a lethality rate of up to 90%. According to national infectious disease surveillance information, WNV infects a significant number of cases globally each year and the mortality rate after infection (~6%) ranks among the highest for infectious disease mortality (Yeung et al., 2017).

Environmental factors such temperature, rainfall patterns, and availability play a crucial role in determining distribution and abundance of mosquito vectors (Ain-Najwa et al., 2020; Fayet, 2020; García-Carrasco et al., 2022). The presence of susceptible bird populations and suitable breeding sites also contribute to WNV transmission. In recent years, there has an increase in WNV in certain regions. This can be attributed to various factors such climate change, urbanization, globalization. Climate change can alter mosquito vectors and bird host geographic range and seasonal activity, potentially expanding the areas risk for WNV transmission. Urbanization can create favorable conditions for mosquito breeding and increase human-mosquito interactions. Globalization facilitates the movement infected individuals or animals across borders, potentially introducing new WNV to different regions (Seidowski et al., 2010; Lu et al., 2014; Fayet, 2020).

Current migratory bird carriage of WNV is difficult to monitor: migratory birds are wild animals and cannot be easily killed. It is difficult to collect tissue samples from migratory birds on a large scale for WNV isolation or detection. Most of the current reports are based on tissue organ sampling of abnormal birds, which makes it difficult to monitor and analyze the scale of WNV infection in detail. In other studies, fecal samples from pigs and penguins can be used for virus detection (Ogrzewalska et al., 2022; Liu et al., 2024). This is all because animals are difficult to sample *in vivo*. Although the virus can be detected in feces (Kipp et al., 2006; Dawson et al., 2007), it is difficult to use feces in actual monitoring.

WNV remains a significant global health concern with its current distribution spanning multiple continents. Looking ahead, it is essential to continue monitoring and studying the epidemiology of WNV to understand its current spread better and predict future trends. This includes surveillance programs detect virus activity in mosquitoes birds, animals, and humans. Additionally, research efforts should be on developing effective vector control strategies and vaccines to prevent mitigate WNV outbreaks. So far, there is no high-throughput detection method applicable to migratory birds for monitoring WNV epidemiology. Traditional detection methods require capturing and dissecting migratory birds. In this study, we established a new WNV monitor method for the detection of viruses carried by migratory birds. This study showed a new perspectives and monitoring methods on the carriage and transmission of WNV by migratory birds, which will be beneficial for WNV prevention and control measures and new vaccine research globally.

## 2 Materials and methods

### 2.1 Sampling and route

**Figure 1:**
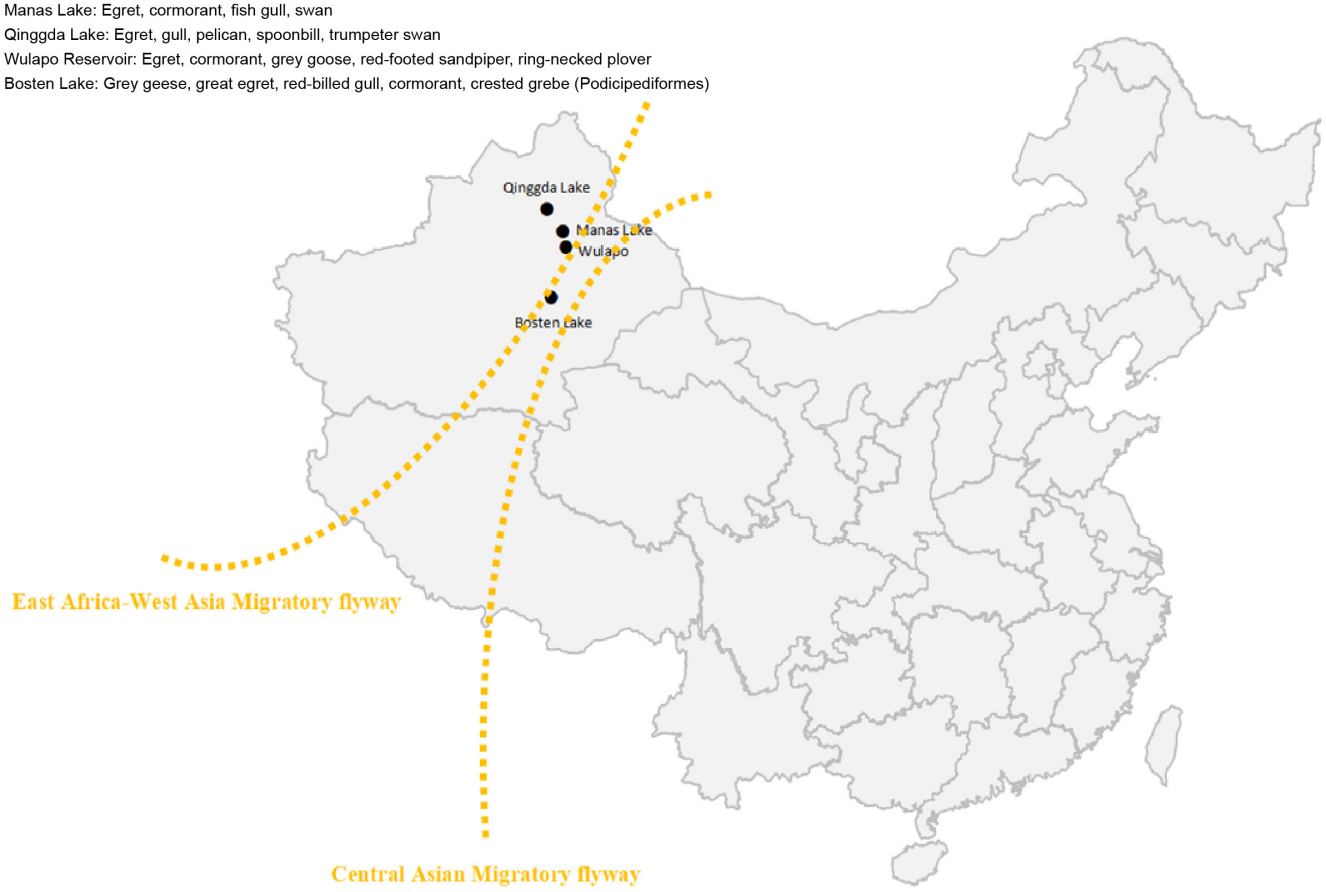
Sampling map. Manas Lake, Bosten Lake, Qinggda Lake, and Wulapo Reservoir in Xinjiang, China were selected as sampling sites. Yellow lines represent the East Africa-West Asia flyway of migratory birds.

The sample collection sites in this study were waypoints located along the migratory routes of migratory birds in the Xinjiang Uygur Autonomous Region of China, namely, Manas Lake, Bosten Lake, Qinggda Lake, and Wulapo Reservoir (Figure 1). Samples were collected in October 2023 and May 2024: 25 samples were collected from Manas Lake and 101 samples were collected from Bosten Lake, totaling 126 samples; and 48 samples were collected from Qinggda Lake and Wulapo Reservoir. One hundred and sixty-one samples were collected from Wulapo Reservoir, and 280 samples were collected from Bosten Lake, totaling 489 samples. The total number of samples collected at the two time points was 515. Samples were collected by placing fresh sticky or solid feces of migratory birds in RNA-free EP tubes, adding RNA preservation solution (Coolabor) in the ratio of 1:1, and storing them for a short period of time at −70°C to −80°C.

### 2.2 RNA extraction

After sampling was completed, the sample RNA was extracted uniformly with the Viral RNA Extraction Kit (QIAGEN Inc.). RNA was isolated according to the manufacturer's instructions with minor adjustments: take 560 μL of the Buffer AVL that had been prepared in a 1.5 mL centrifuge tube. Add 140 μL of sample and vortex for about 15 s. Incubate at room temperature for 10 min. Incubate at room temperature for 10 min. centrifuge briefly to remove the solution from the cap of the tube. Add 560 μL of anhydrous ethanol, vortex for about 15 s, and then centrifuge briefly to remove the solution from the cap. Gently transfer 630 μL of solution from step e to the adsorbent column (in a 2 mL collection tube), centrifuge at 8,000 rpm for 1 min, and transfer the column to a new 2 mL collection tube. Carefully open the adsorbent column and repeat step f until all solutions are filtered by the adsorbent column. Add 500 μL of Buffer AW1 to the column, centrifuge at 8,000 rpm for 1 min, and transfer the column to a new 2 mL collection tube. Add 500 μL of Buffer AW2 to the adsorbent column, centrifuge at the highest speed (14,000 rpm) for 3 min, and transfer the adsorbent column to a new 2 mL collection tube. Centrifuge the column at the highest speed (14,000 rpm) for 1 min, transfer the column to a new 1.5 mL tube, add 60 μL of Buffer AVE, and incubate at room temperature for 1 min. elute RNA: centrifuge at 8,000 rpm for 1 min, and the eluted solution will be the solution containing the sample RNA.

### 2.3 Amplicon design and PCR detection

**Figure 2:**
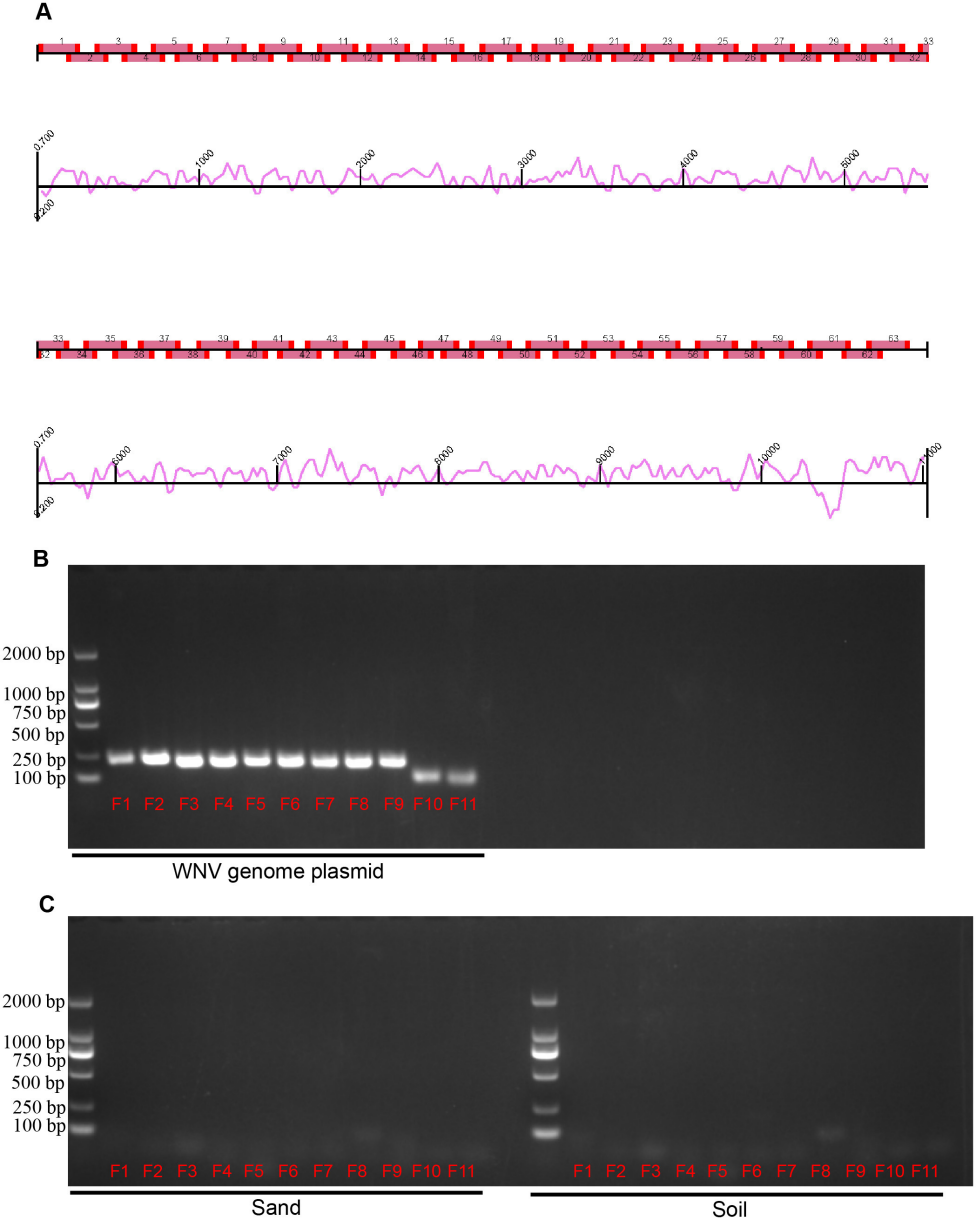
Design and validation of West nile virus amplicons. **(A)** Map of WNV amplicons. Sixty-three amplicons covering the WNV genome, with curves indicating the potential efficiency of amplification. **(B)** Validation of amplicon primers by WNV genome plasmid. **(C)** Validation of amplicon primers by environmental factors. Sand and soil in sampling sites were detected. Lane 1 (from left to right) is DNA marker, lanes 2–12 (F1–F11) are fragments of the amplicon.

For WNV RNA amplication, amplicon methods were employed (Song et al., 2022). By using the NY99 strain WNV genome (DQ211652) as the reference sequence, a total of 63 amplicons of viral genes were designed (Figure 2). Based on the full-length genome sequence of reference virus, we designed a total of 63 pairs of primers for the amplification of the viral nucleic acid. Each amplification covered a viral fragment of about 200 bp, and each individual fragment has an overlap of about 30 bp in length. The 63 pairs of primers cover the full length of the WNV virus genome. After sequencing the amplified fragments, the residual components of the WNV nucleic acid in the sample can be determined.

In order to test all samples, every 25–30 fecal samples were combined into one test group. From a total of 615 fecal samples of migratory birds collected in Xinjiang, China, of which 126 were collected in 2023 and 489 in 2024, total RNA was extracted using microsample extraction method and then analyzed via amplification and sequencing. The collected migratory bird fecal samples included undried fresh guano and dried guano (presumed to be no more than 2 weeks old based on the degree of drying and weathering). The fragments obtained via amplification were purified, cloned to the T vector, and sequenced, and the sequencing results were compared and analyzed in NCBI database. Finally, 11 amplicons of the viral genes were detected, and these 11 amplicons with serial number designations and sequence information are listed in Tables 1, 2.

**Table 1:** Sequences of WNV amplicon.

**No**.	**Sequences**	**Length**
1	GGTGGGAAAACCCCTGCTCAACTCAGACACCAGTAAAATCAAGAACAGGATTGAACGACTCAGGCGTGAGTACAGTTCGACGTGGCACCACGATGAGAACCACCCATATAGAACCTGGAACTATCACGGCAGTTATGATGTGAAGCCCACAGGCTCCGCCAGTTCGCTGGTCAATGGAGTGGTCAGGCTCCTCTCAAAACCAT	203
2	CACTCGCACCACCACAGAGAGCGGAAAGTTGATAACAGATTGGTGCTGCAGGAGCTGCACCTTACCACCACTGCGCTACCAAACTGACAGCGGCTGTTGGTATGGTATGGAGATCAGACCACAGAGACATGATGAAAAGACCCTCGTGCAGTCACAAGTGAATGCTTATAATGCTGATATGATTGACCCTTTTCAGTTGGGC	205
3	GAACAACAGATCAATCACCATTGGCACAAGTCTGGAAGCAGCATTGGCAAAGCCTTTACAACCACCCTCAAAGGAGCGCAGAGACTAGCCGCTCTAGGAGACACAGCTTGGGACTTTGGATCAGTTGGAGGGGTGTTCACCTCAGTTGGGAAGGCTGTCCATCAAGTGTTCGGAGGAGCATTCCGC	186
4	CTCGTGGGCTGCTCGGCAGTTATCAAGCAGGAGCGGGCGTGATGGTTGAAGGTGTTTTCCACACCCTTTGGCATACAACAAAAGGAGCCGCTTTGATGAGCGGAGAGGGCCGCCTGGACCCATACTGGGGCAGTGTCAAGGAGGATCGACTTTGTTACGGAGGACCCTGGAAATTGCAGCACAAG	185
5	ATCACAGAATACACCGGGAAGACGGTTTGGTTTGTGCCTAGTGTCAAGATGGGGAATGAGATTGCCCTTTGCCTACAACGTGCTGGAAAGAAAGTAGTCCAATTGAACAGAAAGTCGTACGAGACGGAGTACCCAAAATGTAAGAACGATGATTGGGACTTTGTTATCACAACAGACATATCTGA	185
6	CCTGATCGACGGCAAGGGGCCAATACGATTTGTGTTGGCTCTCTTGGCGTTCTTCAGGTTCACAGCAATTGCTCCGACCCGAGCAGTGCTGGATCGATGGAGAGGTGTGAACAAACAAACAGCGATGAAACACCTTCTGAGTTTTAAGAAGGAACTAGGGACCTTGACCAGTGCTATCAATCGG	184
7	ACCTGGCAAGAACGTTAAGAACGTCCAGACGAAACCAGGGGTGTTCAAAACACCTGAAGGAGAAATCGGGGCCGTGACTTTGGACTTCCCCACTGGAACATCAGGCTCACCAATAGTGGACAAAAACGGTGNNGTGATTGGGCTTTANGGCAATGGAGTCATAATGCCCAACGGCTCAT	179
8	GAAAGTCATAGAGAAGATGGAGCTGCTCCAACGCCGGTATGGGGGGGGACTGGTCAGAAACCCACTCTCACGGAATTCCACGCACGAGATGTATTGGGTGAGTCGAGCTTCAGGCAATGTGGTACATTCAGTGAATATGACCAGCCAGGTGCTCCTAGGAAGAATGGAAAAAAGGACC	178
9	CTGAGGAAAAAACAGATCACTGTACTGGATCTCCATCCCGGCGCCGGTAAAACAAGGAGGATTCTGCCACAGATCATCAAAGAGGCCATAAACAGAAGACTGAGAACAGCCGTGCTAGCGCCAACCAGGGTTGTGGCTGCTGAGATGGCTGAAGCACTGAGAGGACTGCCCATCC	175
10	GTAAAACAAGGAGGATTCTGCCACAGATCATCAAAGAGGCCATAAACAGAAGACTGAGAACAGCCGTGCTAGCGCCAACCAG	82
11	AAAGCTGCGTGCCCGACCATGGGAGAAGCTCACAATGACAAACGTGCTGACCCAGCTTTTGTGTGCAGACAAGGA	75

**Table 2:** Primers of WNV genome amplicon.

**Number of amplicons**	**Sequences of primers**
1	F: GGTGGGAAAACCCCTGCTCA
	R: ATGGTTTTGAGAGGAGCCTG
2	F: CACTCGCACCACCACAGAGA
	R: GCCCAACTGAAAAGGGTCAA
3	F: GAACAACAGATCAATCACCA
	R: GCGGAATGCTCCTCCGAACA
4	F: CTCGTGGGCTGCTCGGCAGT
	R: CTTGTGCTGCAATTTCCAGG
5	F: ATCACAGAATACACCGGGAA
	R: CAGATATGTCTGTTGTGATA
6	F: CCTGATCGACGGCAAGGG
	R: CCGATTGATAGCACTGGT
7	F: ACCTGGCAAGAACGTTA
	R: ATGAGCCGTTGGGCATTA
8	F: GAAAGTCATAGAGAAGATGG
	R: GGTCCTTTTTTCCATTCTTC
9	F: CTGAGGAAAAAACAGATCAC
	R: GGATGGGCAGTCCTCTC
10	F: GTAAAACAAGGAGGATTCTG
	R: CTGGTTGGCGCTAGCA
11	F: AAAGCTGCGTGCCCGACC
	R: TCCTTGTCTGCACACAAA

The micro-extracted sample RNA was prepared according to the following table as a reverse transcription system, gently mixed, and then reacted in a PCR instrument through the reverse transcription program: 37°C for 15 min, 85°C for 5 s. After the end of the reaction, the reaction product was temporarily placed at 4°C for storage. After reverse transcription, the PCR system was prepared according to the following table, gently mixed, and then the reaction program in the PCR instrument was as follows: pre-denaturation at 94°C for 5 min, denaturation at 94°C for 30 s, annealing at 55°C for 30 s, extension at 72°C for 15 s, and final extension at 72°C for 10 min, with denaturation-annealing for 30 cycles. The full-length WNV genomic (DQ211652) was synthesized by Synbio company and cloned in pACYC177 plasmid in our laboratory. Purified plasmids were used as positive controls. Soil and sand in the sampling environment were used as negative controls.

PCR products were validated using electrophoresis by running 20 μL of PCR product with 10X loading buffer for 30 min. Bands of each amplicons were evaluated by comparing with DL2000 DNA marker. Correctly sized bands were cut and sequenced.

## 3 Results

**Figure 3:**
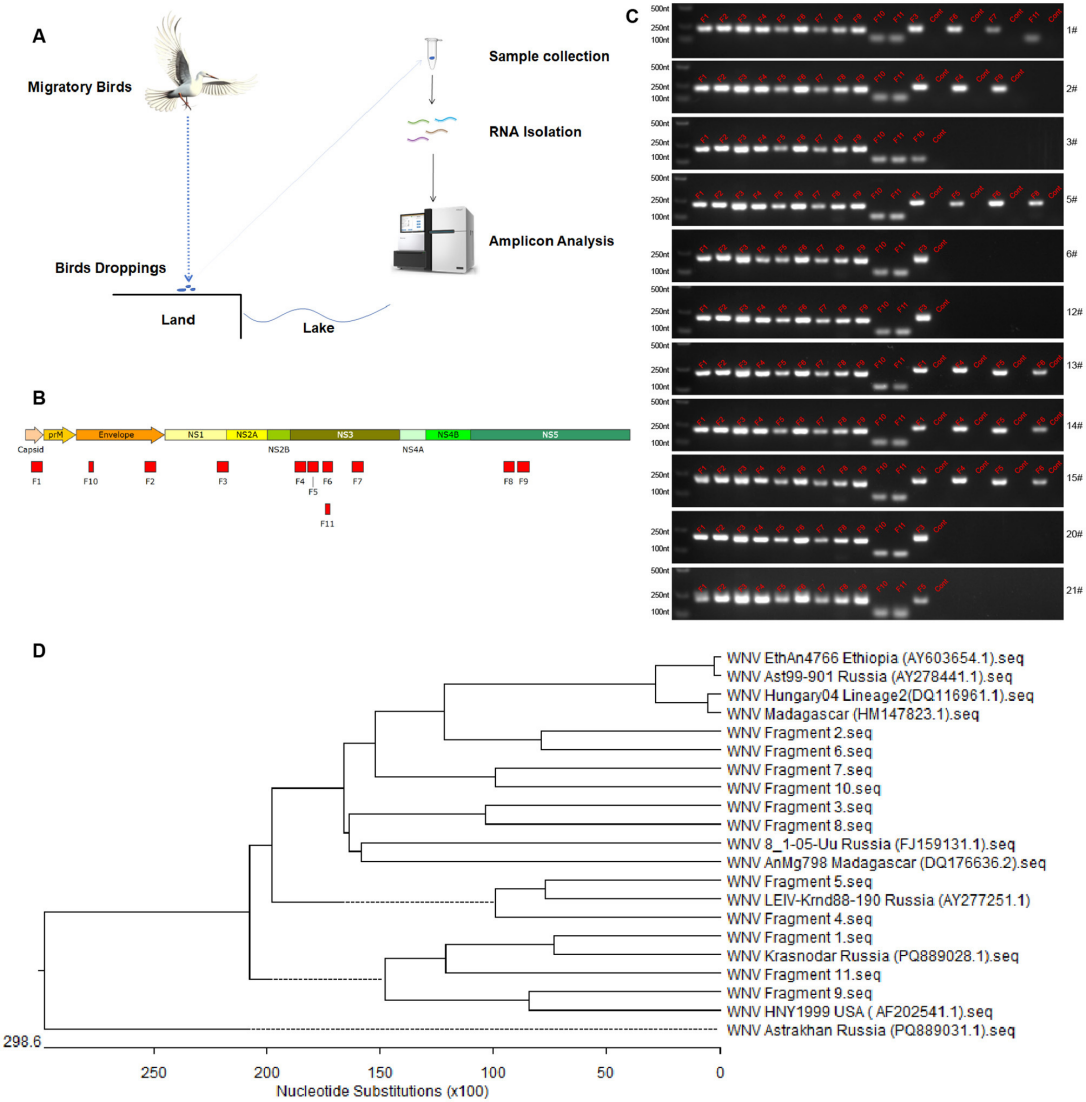
Detection of WNV fragments in feces of migratory birds. **(A)** Schematic diagram of sample collection and viral fragments detection. **(B)** Amplicons obtained in the whole viral genome amplification. Eleven amplicons from viral gene were determined by amplification and sequencing. **(C)** Electrophoretic analysis of WNV fragments. Lane 1 (from left to right) is DNA marker, lanes 2–12 are fragments of the amplicon obtained from WNV plasmid as positive control, other lanes are amplicons obtained from feces samples or negative controls (cont). The numbers of samples are presented on the right of each electrophoretic image. **(D)** Phylogenetic analysis of WNV fragments. The phylogenetic analysis was performed using the R code.

Effective monitoring of WNV carriage by migratory birds is difficult. In this study, we developed a method for detecting WNV nucleic acids in bird dropping samples via amplification and analysis of WNV genome fragments and detected WNV in fecal samples of migratory birds collected on different years (2023 and 2024) in Xinjiang, China, which proved that the source of WNV infection risk in Xinjiang, China, is related to migratory birds (Figure 3). Based on the genome sequence of the NY99 strain of WNV (National Center for Biotechnology Information accession no. DQ211652.1), we divided the WNV genome sequence into 63 amplicons (Figure 3). Each amplicon was ~200 nt in length, with ~50 nt of overlapping sequences between adjacent amplicons. The primers for each amplicon were 20 nt in length (primers available upon request).

Analysis of the migratory bird fecal samples showed that 101 of the 126 and 200 of the 489 migratory bird fecal samples collected in 2023 and 2024, respectively, were WNV nucleic acids positive, with 11 and 5 WNV gene fragments amplified via amplicon sequencing, respectively (Table 3). The detection rate of WNV nucleic acids in migratory bird feces ranges from 39% to 41%. The five gene fragments in the 2024 samples were included in the 11 gene fragments in the 2023 samples and ranged from 80 to 200 nt in length. Sequencing data proved that the 11 gene fragments obtained belonged to WNV.

**Table 3:** WNV fragments detection in fecal samples.

**Groups**	**Fecal samples in each groups*a***	**Fragment of viral RNA**	**Number of RNA fragments**	**Sampling point*b***	**Sampling time**
1#	25	3, 6, 7, 11	4	Manas Lake	2023.10
2#	25	2, 4, 9	3	Bosten Lake	2023.10
3#	25	10	1	Bosten Lake	2023.10
4#	25	None	None	Bosten Lake	2023.10
5#	26	1, 5, 6, 8	4	Bosten Lake	2023.10
6#	50	3	1	Ulanbuy	2024.05
7#	50	None	None	Ulanbuy	2024.05
8#	30	None	None	Ulanbuy	2024.05
9#	31	None	None	Ulanbuy	2024.05
10#	24	None	None	Qinggeda Lake	2024.05
11#	24	None	None	Qinggeda Lake	2024.05
12#	25	3	1	Bosten Lake	2024.05
13#	25	1, 2, 4, 5	4	Bosten Lake	2024.05
14#	25	1, 2, 4, 5	4	Bosten Lake	2024.05
15#	25	1, 2, 4, 5	4	Bosten Lake	2024.05
16#	25	None	None	Bosten Lake	2024.05
17#	25	None	None	Bosten Lake	2024.05
18#	25	None	None	Bosten Lake	2024.05
19#	25	None	None	Bosten Lake	2024.05
20#	25	3	1	Bosten Lake	2024.05
21#	25	5	1	Bosten Lake	2024.05
22#	30	None	None	Bosten Lake	2024.05

^a^25–50 fecal samples were combined into one group.

^b^All samples were collected in Xinjiang, China.

Comparison of these 11 gene fragments revealed that five of them were from NS3, two were from NS5, two were from E, and one each was contained in NS1 and the capsid. Nucleic acid fragments from NS3 had the highest abundance and detection rates and were detected in both the 2023 and 2024 samples. In order to analyze the genetic relationships of WNV fragments, phylogenetic analysis was performed (Figure 3D). The results showed that the fragments from Manas Lake were closely related to HM147823.1 and FJ159131.1. Only one fragment was detected in Ulanbuy, which was closely related to FJ159131.1. All fragments except Fragment seven were detected in Bosten Lake, with relatively loose genetic relationships. This indicates that the viruses carried by migratory birds do not have obvious strain—specific characteristics. By comparing the strains from East Africa and Russia, it can be seen that the WNV strains in the countries along these migratory bird routes do not show obvious regional distribution characteristics, and all contain strains closed to Lineage 1 (AF202541.1) and Lineage 2 (DQ116961.1).

The sampling sites were different lakes in Xinjiang: Manas Lake and Bosten Lake for the 2023 samples, and Wulapo Reservoir, Qinggeda Lake and Bosten Lake, which are the most active sites for migratory birds, for the 2024 samples. WNV nucleic acids were detected in samples from all locations, except Qinggeda Lake. The sampling time was when migratory birds in Xinjiang concentrated their activities: the 2023 sampling was in October, and the 2024 sampling was in May. These were the most active times for migratory birds in Xinjiang and the time when mosquitoes were most active. The interior of Xinjiang is covered by the East Africa-West Asia migratory flyway and the Central Asian migratory flyway, both of which pass through regions where WNV is prevalent lakes such as Manas Lake and Bosten Lake in Xinjiang are stopover or roosting sites for migratory birds, and a large number of migratory birds and bird dropping samples can be found and collected at these sites. Therefore, collecting fecal samples of birds from these locations can help determine the risk of carrying the WNV in the East Africa-West Asia migratory flyway and the Central Asian migratory flyway.

## 4 Discussion

A small number of WNV infections were reported in Xinjiang, China, between 2005 and 2014, and some cases may have been unaccounted for or undetected (Li et al., 2013; Lu et al., 2014; Cao et al., 2017; Zhang et al., 2021). The reason is WNV pathogens are not conventional measured in the non-endemic region. A clue to the discovery of these infections was the presence of patients with fever or viral encephalitis in local hospitals, and these infections were experimentally diagnosed via detection of WNV IgM antibodies or WNV-neutralizing antibodies in the serum. WNV nucleic acids have also been isolated from mosquitoes in Xinjiang, and primers designed for the WNV E and NS5 genes have been used to detect WNV nucleic acids in a subset of mosquitoes (Lu et al., 2014; Cao et al., 2017). These data suggest that mosquitoes in the Xinjiang region carry the WNV and can cause human infections. Five strains of WNV were isolated from mosquitoes that tested positive, and sequencing of these strains showed that they belong to lineage 1, which is highly homologous to the WNV strains prevalent in Russia (Cao et al., 2017). This suggests that the WNV cases in Xinjiang, China, may be related to bird migratory, as migratory routes connect WNV-endemic regions in Eastern Europe and Africa and passes through Xinjiang, China, and Russia.

Effective monitoring of WNV carriage by migratory birds using current technological methods is difficult (Seidowski et al., 2010; Ain-Najwa et al., 2020), which makes infection risk analysis and prediction of WNV epidemics difficult. A new convenient and rapid assay is important for WNV nucleic acid surveillance and risk assessment. Based on our new test method, it is possible to determine whether migratory birds carry or have carried the WNV virus, and to determine the regional transmission and epidemiological risk of WNV based on the carriage of viral nucleic acids by migratory birds.

In our experiment, the amplicon detection method was introduced, and 63 pairs of primers were designed to cover the full-length genome of the West Nile virus (WNV). Compared with the conventional PCR detection, this method will not miss the viral nucleic acid in the samples. It can avoid the problem of false negatives caused by RNA degradation and is suitable for the detection of fecal samples. It should be noted that this method is more likely to produce false positives during the amplification process, so it is necessary to sequence and verify the amplified gene fragments. Considering the difficulty of experiments on migratory birds, using the amplicon method to detect viral nucleic acid in feces is a good choice for monitoring viral nucleic acid in migratory birds. Detection of WNV nucleic acids in the feces of migratory birds allows speculation on whether the bird is carrying WNV virus (Liu et al., 2024), but does not provide valid information on the extent of carriage and tissue and organ deficiencies. Further experimental studies are needed to obtain detail infection information and bird species information from feces.

China has not historically been an endemic area for WNV, and only occasional cases have been reported in Xinjiang. Based on the flight routes of migratory birds, the occurrence of WNV in Xinjiang may be related to the migration of birds on the East Africa-West Asia migratory flyway and the Central Asian migratory flyway; however, no relevant study has confirmed this hypothesis (Fayet, 2020; Ferraguti et al., 2024). Migratory bird feces are easy to collect and do not harm migratory birds; however, their RNA is easily degraded (Kagzi et al., 2022). Despite this, RNA degrades over time, and some are not degraded quickly or completely because of their structure and external environment. To the best of our knowledge, this study is the first to determine that migratory birds in Xinjiang carry the WNV via microextraction of RNA from feces of migratory birds and subsequent detection of WNV amplicons. The detection rate of WNV nucleic acids in the feces of fresh and 2-week-old migratory birds ranged from 39% to 41%, suggesting that the WNV carriage rate of migratory birds was not <39%. The results of this study are important for WNV epidemic prediction, prevention, and control, and provide a new method for the global monitoring of WNV carriage in migratory birds.

## Data Availability

The original contributions presented in the study are included in the article/supplementary material, further inquiries can be directed to the corresponding authors.
