# Correction: Evidence of WNV infection in migratory birds passing through Xinjiang, china, using viral genome amplicon approach

**DOI:** 10.3389/fmicb.2025.1638113

**Published:** 2025-06-13

**Authors:** Kunsheng Tao, Chan He, Tong Zhang, Changguang Xiao, Lifei Du, Zongjie Li, Donghua Shao, Jianchao Wei, Beibei Li, Yafeng Qiu, Zhiyong Ma, Ke Liu

**Affiliations:** ^1^Shanghai Veterinary Research Institute, Chinese Academy of Agricultural Science, Shanghai, China; ^2^Hunan Institute of Animal and Veterinary Science, Changsha, China

**Keywords:** West Nile virus, migratory bird, amplicon, fecal samples, epidemiology

In the published article, there was an error in the legend for Figure 3D as published. [**(D)** Phylogenetic analysis of WNV fragments. The phylogenetic analysis was performed using the DNAstar software.]. The corrected legend appears below.

[**(D)** Phylogenetic analysis of WNV fragments. The phylogenetic analysis was performed using the R code.].

The authors apologize for this error and state that this does not change the scientific conclusions of the article in any way. The original article has been updated.

