# Supplementary figures and images for: Evidence of WNV infection in migratory birds passing through Xinjiang, China, using viral genome amplicon approach
Source: Front Microbiol. 2025 Mar 26;16:1468530. doi: 10.3389/fmicb.2025.1468530 (PMC11979221; doi:10.3389/fmicb.2025.1468530)

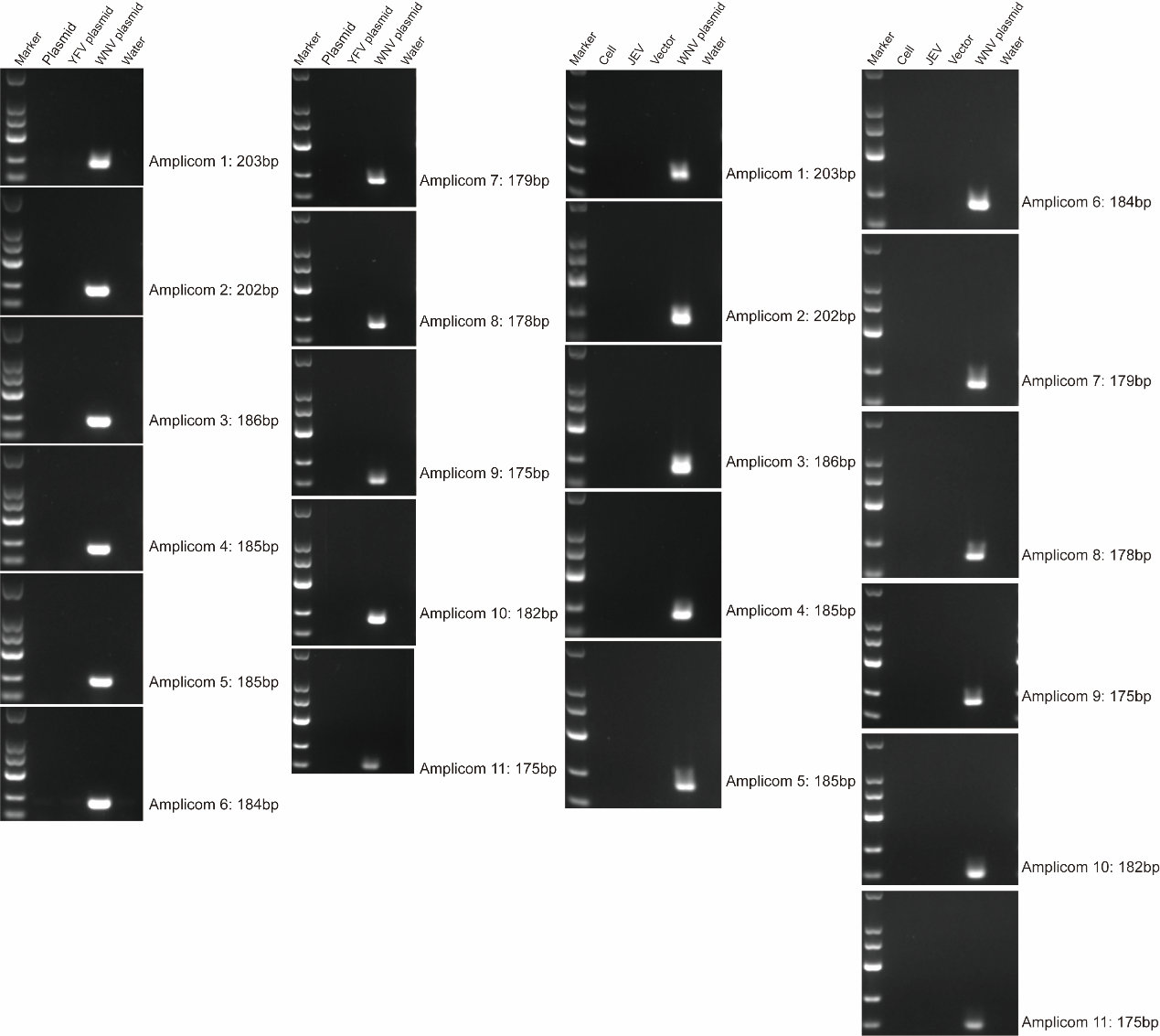

Supplement: Supplementary file 1 [file Image_1.tif]

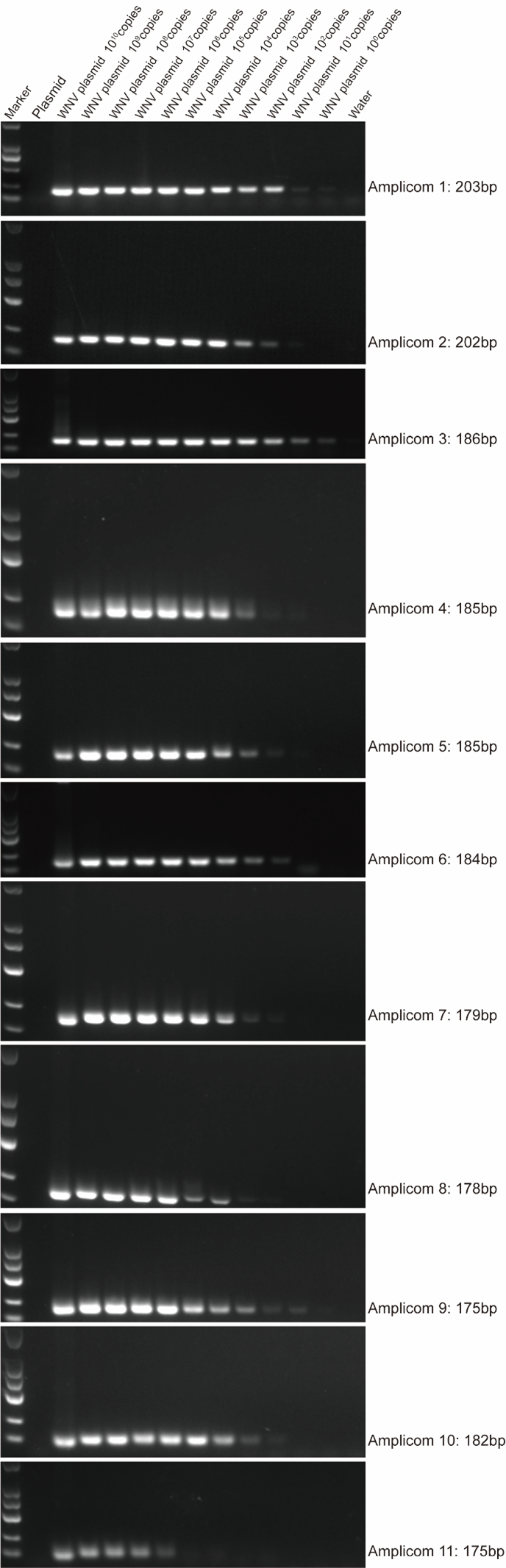

Supplement: Supplementary file 2 [file Image_2.tif]
